# An ideal biomaterial: Ti-based bone repair material with dual-response antibacterial system and sustained drug release

**DOI:** 10.3389/fbioe.2025.1619084

**Published:** 2025-09-02

**Authors:** Wentao Zhang, Zhaoyang Guo, Zhongyuan He, Hang Zhou, Hang Liu, Xinliang Peng, Xingyu Yang, Ru Zhong, Wen Huang, Lei Chu, Zhongliang Deng

**Affiliations:** ^1^ Department of Orthopaedics, The Second Affiliated Hospital of Chongqing Medical University, Chongqing, China; ^2^ National Engineering Research Center for Tissue Restoration and Reconstruction, South China University of Technology, Guangzhou, China

**Keywords:** bone infection, antibacterial, electronic biological response, enzymatic hydrolysis response, bone repair, titanium-based materials

## Abstract

Bone infection is a disease with high treatment cost, long period and high disability rate in orthopedics. The development of antibacterial implant materials *in vivo* can alleviate the pain of patients and social burden. How to make the antibacterial substances in the implant materials release regularly as needed is a key technical breakthrough that antibacterial bone repair implants have been trying to optimize. It has been proved that metal silver ions sputtering can form higher specific surface area on the surface of implanted materials, and can produce electron biological effect to realize *in situ* sterilization. Antibacterial peptide crosslinked with hyaluronic acid can be hydrolyzed by hyaluronidase during bacterial infection, thus killing free bacteria, producing immune regulation. At present, it is planned to use titanium dioxide nanotubes to construct a double-layer nanotube structure, fill osteogenic drugs into the nanotubes by vacuum-assisted physical adsorption, and sputter deposit metal silver ions on the surface of the nanotubes. The outer layer of the material is prepared by covalent grafting of antibacterial peptides and hyaluronic acid to prepare a layer-by-layer assembly technology shell to prepare a bone implant scaffold material with dual sterilization response systems of electronic biological antibacterial response and enzymatic hydrolysis antibacterial response.

## Introduction

After the aging of the population, the problem of bone injury caused by external violence and internal inflammation, tumor, osteoporosis and other diseases is becoming more and more serious. Regeneration and repair after bone injury is a complex physiological process including reconstruction and replacement of damaged tissue.

From the occurrence of bone injury to the final healing, the whole process can be roughly divided into three stages: inflammation stage, repair stage and remodeling stage. Although the repair can be divided into three stages according to the main events during the repair of bone injury, these three stages are not strictly distinguished and do not exist completely independently. As a continuous process, these events will occur simultaneously at the junction in different stages of bone injury repair (Wang et al., 2020; Claes et al., 2012). For example, when the inflammatory period and the repair period are in transition, although the activity of immune cells has been relatively reduced, the gradually accelerated proliferation of osteoblasts in the repair is still accompanied by the cellular behavior of immune cells to help repair various damaged tissues at the injury site. To sum up, the implantation of bone repair materials and their corresponding functions in different periods will affect the bone repair process in different periods and produce different effects.

With the aging population and the increasing incidence of metabolic diseases related to bone repair, such as diabetes, the application and demand of various bioactive materials in bone defect repair-related surgery are increasing, and the types of materials that can be used to promote bone repair and regeneration are also increasing (Guo et al., 2023; Saul and Khosla, 2022). Appropriate bioactive materials can increase the adhesion, differentiation and proliferation of cells by combining with macromolecules, cells and other elements, and finally promote bone repair and regeneration (Tang et al., 2021; Sovova et al., 2021). Bone repair materials lacking biological activity are at risk of bacterial infection after implantation, and also face the problem of poor bone integration (Table 1) (Jacobs et al., 2000; Porter et al., 2021; Ingham and Fisher, 2005; Pacheco, 2019; Zhang et al., 2023; Bäcker et al., 2023). In the failure cases of implant materials, 25% cases were caused by bacterial infection, and 16% cases were caused by loose implant materials (Bozic et al., 2010). For implant surgery, bacterial infection is a very serious clinical problem. When bacterial infection occurs at the implant site, it will cause a series of serious complications for patients, and may even lead to sepsis. Poor osseointegration will loosen the implant material, which is also the key factor to cause the failure of the implant material (Raphel et al., 2016). In addition, it is found that when bacteria are infected, the environment of inflammation and bone absorption will lead to the wear of implant materials and produce particles, which will lead to poor bone integration. However, the surface of implant materials with poor osseointegration is more likely to adhere to bacteria, which leads to more bacteria proliferation on the surface and more serious bacterial infection (Costerton et al., 1999). These two factors cooperate with each other to further improve the failure rate of implant materials. Therefore, designing functional implant materials that can prevent bacterial infection and promote bone integration is the key to successful surgery.

**TABLE 1:** Main problems faced by clinical orthopedic implants after implantation.

Types	Reasons	Improvement measures	References
Neurovascular injury, hemorrhage and hematoma	Intraoperative nerve injury, intraoperative bleeding to form hematoma compression nerve	Intraoperative navigation or nerve monitoring, complete hemostasis during operation	Jacobs et al. (2000)
Infection associated with implants	Operation pollution, bacterial biofilm formation	Sterile operation, antibiotics before and after operation, and antibacterial coating on implants	Porter et al. (2021)
Aseptic loosening	Wear debris activates macrophages and causes osteolysis	Use low-wear materials and anti-bone absorption drugs	Ingham and Fisher (2005)
Metal ion release and allergy	Ion release leads to local/systemic toxicity	Using inert materials, patch test before operation	Pacheco (2019)
Stress shielding and bone atrophy	Rigid implants lead to stress shielding and local osteoporosis	Using degradable materials, the materials are designed with gradient modulus	Zhang et al. (2023)
Mechanical failure (fracture, displacement)	Fracture of bone plate, spinal screw cutting vertebral body	3D printed personalized prosthesis	Bäcker et al. (2023)

Porous structure can make tissues and cells grow, adhere and maintain cell activity in the material space, and even form osteon structure, which is beneficial to enhance the stable combination of materials and body tissues and avoid the problems of loosening and falling off after transplantation (Sovova et al., 2021; Dec et al., 2022). Scaffold material is an important center of tissue engineering bone. The ideal scaffold needs to simulate the three-dimensional structure of extracellular matrix (ECM), and the best bone repair material should have the following advantages: Firstly, the scaffold needs to own good biocompatibility to support the adhesion and proliferation of osteoblasts. Secondly, it must have good mechanical properties. At the same time, it is necessary to have pore connectivity suitable for transporting nutrients and oxygen. Moreover, it should also meet the requirements of signal molecules that are conducive to the directional differentiation of cells into ideal types of morphology, so as to promote cell adhesion, proliferation, metabolism and differentiation. It should also have the function of promoting vascularization to meet the nutritional supply and waste removal of tissue growth. Degradable scaffold materials also need to meet the biodegradability or bioresorbability, so as to provide growth space for new bone tissue (Li et al., 2017; Alghamdi and Jansen, 2020).

Inorganic materials include metallic materials and nonmetallic materials. Compared with natural materials, metal materials are widely used in bone repair because of their excellent hardness and stiffness mechanical properties, especially when the bone tissue to be repaired still needs to bear strong pressure. Available metal materials include titanium (Ti), tantalum, cobalt, magnesium alloy, etc (Niinomi, 2008; Elias et al., 2008; Albrektsson and Johansson, 2001). When pure metal is transplanted into the body bone environment, it will face some problems, such as insufficient flexibility, corrosion of metal body, failure to form a stable link with bone tissue cells, *etc.* In order to solve these pain points, methods such as coating alloy materials with metal surface is easy to establish tissue connectors have been developed. Bioactive materials can promote the rate and efficiency of inflammatory reaction, bone formation, angiogenesis and other processes in bone injury repair by influencing immune cells, bone formation-related cells, osteoblast progenitor cells, vascular endothelial cells and so on (Wei et al., 2023; Zhao et al., 2023). At present, a large number of studies on Ti materials show that the rough surface of Ti can improve the integration of exogenous implant materials and bulk bone tissue, and promote the osteogenic differentiation of mesenchymal stem cells (MSCs) compared with smooth surfaces (Vermeulen et al., 2022; Solier et al., 2023; Buser et al., 2004). The regulation of cell differentiation ability by surface roughness treatment of this metal material may be realized by directly changing cell shape and adhesion state and further affecting integrin signaling pathway (Dalby et al., 2007). Anodizing is the most commonly used way to modify Ti-based surface at present. Anodizing can form titanium dioxide (TiO_2_) nanotubes on Ti and its surface. By adjusting the time and voltage of anodizing, a multi-layer nanotube structure can be constructed on Ti-based surface.

Although artificial bone repair materials have the advantages of flexible design and considerable output, there are still some problems such as lack of activity and insufficient bone conductivity. The research and development of ideal artificial bone replacement materials is still one of the main challenges facing clinical and basic research in orthopedics (Bharadwaz and Jayasuriya, 2020; Bashir et al., 2023). At present, it is the preferred strategy in the field of bone tissue engineering to combine various materials and combine their advantages to prepare scaffolds with biological activity, suitable porosity and excellent mechanical properties. Growth factors play an important regulatory role in the process of bone formation, reconstruction and regeneration, mainly focusing on enhancing the biological functions of bone grafts, such as the recruitment of endogenous MSCs, endothelial cell migration and osteogenic differentiation (Martino et al., 2015). Different growth factors are needed in different stages of bone regeneration, so different kinds of growth factors can be loaded in different materials and play a role in time, thus playing a role in specific stages, simulating the bone regeneration process more accurately and obtaining better functional performance *in vivo*. The controlled release of drugs and bioactive substances plays an important role in bone defect repair, and it is particularly critical to realize controlled and sustainable delivery through reasonable material structure design, which can be combined with bioactive factors to promote bone repair.

At present, the clinically approved orthopedic antibacterial implant system mainly covers joint replacement, trauma fixation and spinal fusion, and its antibacterial mechanism involves antibacterial coating, sustained release of antibiotics and material modification. Antibiotic joint cement can slowly release antibiotics and reduce the risk of postoperative infection (Xu et al., 2020). Antibacterial coating orthopedic implants can play a role through material surface coating and inhibit biofilm formation through contact sterilization (Akay and Yaghmur, 2024). Absorbable antibacterial internal fixation system can continuously release antibacterial components through degradation process (Lin Z. et al., 2019). The composite antibacterial bone repair material can combine drug loading and antibacterial, and has both bone conduction and antibacterial functions (Li et al., 2022). Among them, the currently approved antibacterial implants are mainly antibiotic bone cement and antibacterial coated metal implants. The extensive use of antibiotics in orthopedic perioperative period can easily lead to bacterial resistance and increase the economic burden of patients (Parvizi et al., 2010). Therefore, it is the pursuit and vision of many scientists at home and abroad to develop antibacterial bone repair materials through non-antibiotic routes. At present, non-antibiotic loaded bone repair materials mainly use composite inorganic antibacterial agents, composite organic antibacterial agents and bionic nano-structured surface antibacterial agents to achieve antibacterial function. The growth rate of bone tissue is slow, so it is challenging to treat bone defects and other related diseases through self-repair. The risk of bacterial infection in bone injury is as high as 15%–55% (Morgenstern et al., 2018), while the infection rate of bone joint implantation can reach 4.9% (Kazimierczak et al., 2022). More serious bone infection even requires amputation. The most effective way to solve this problem is that bone repair materials have antibacterial properties themselves.

After biomaterials are implanted into the body, biological cascade reactions such as bacterial adhesion-biofilm formation and cell adhesion-proliferation-differentiation-tissue formation all occur at the material interface (Raphel et al., 2016; Kazimierczak et al., 2022; Kaneko et al., 2021; Busscher et al., 2012). The interface design of material is very important for the research and development of biomaterials and implant equipment. The adhesion of bacteria and cells on the surface of implants is associated and competitive. Gristina put forward the theory of “race for the surface”, pointing out that bacteria and cells compete for living space on the surface of implants (Gristina, 1987). If the host cells can reach and occupy the implant surface, it can not only achieve stronger tissue integration at first, but also establish a defense barrier to resist microbial adhesion and reproduction. After bacteria adhere and stabilize, biofilm will form within 12–18 h, leading to implant infection. In the process of biofilm formation, bacterial colonies will form and secrete polysaccharide layers to protect them from host immune response and attack of therapeutic drugs. The dosage of antibiotics required to kill bacteria in biofilm is 1,000 times higher than that required to kill suspended/initial adhesion bacteria (Hetrick and Schoenfisch, 2006). Inhibition of initial bacterial adhesion is the core of antibacterial implant design. It is very important to optimize the implant interface design under the guidance of the core, endow the implant with excellent antibacterial performance and promote the integration of the implant with surrounding bone.

The ideal graft material should have osteoinductivity, osteoconductivity and osseointegration, good biocompatibility and biodegradability, and meet the requirements of mechanical properties as well as biological properties (Holzapfel et al., 2017). A single material is compounded into a composite material with better mechanical properties by bioengineering technology, and the prepared biological scaffold is supplemented with cytokines to improve its biological properties, which can greatly meet the microenvironment requirements of bone regeneration (Cunniffe et al., 2017).

## Hypothesis

**FIGURE 1:**
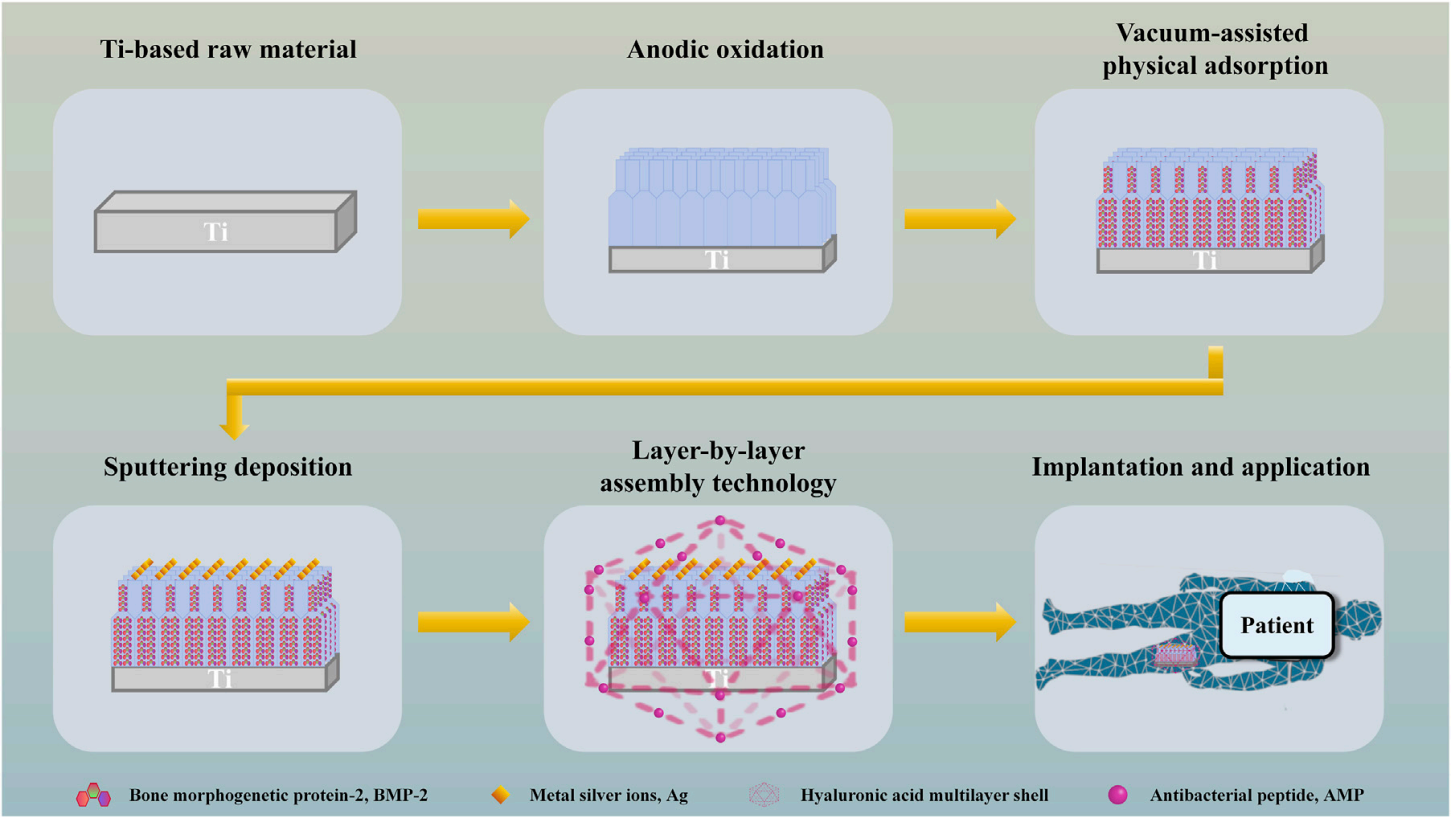
Schematic depiction of the preparation and application of bone implant scaffold with dual sterilization response systems of electronic biological antibacterial response and enzymatic hydrolysis antibacterial response.

**FIGURE 2:**
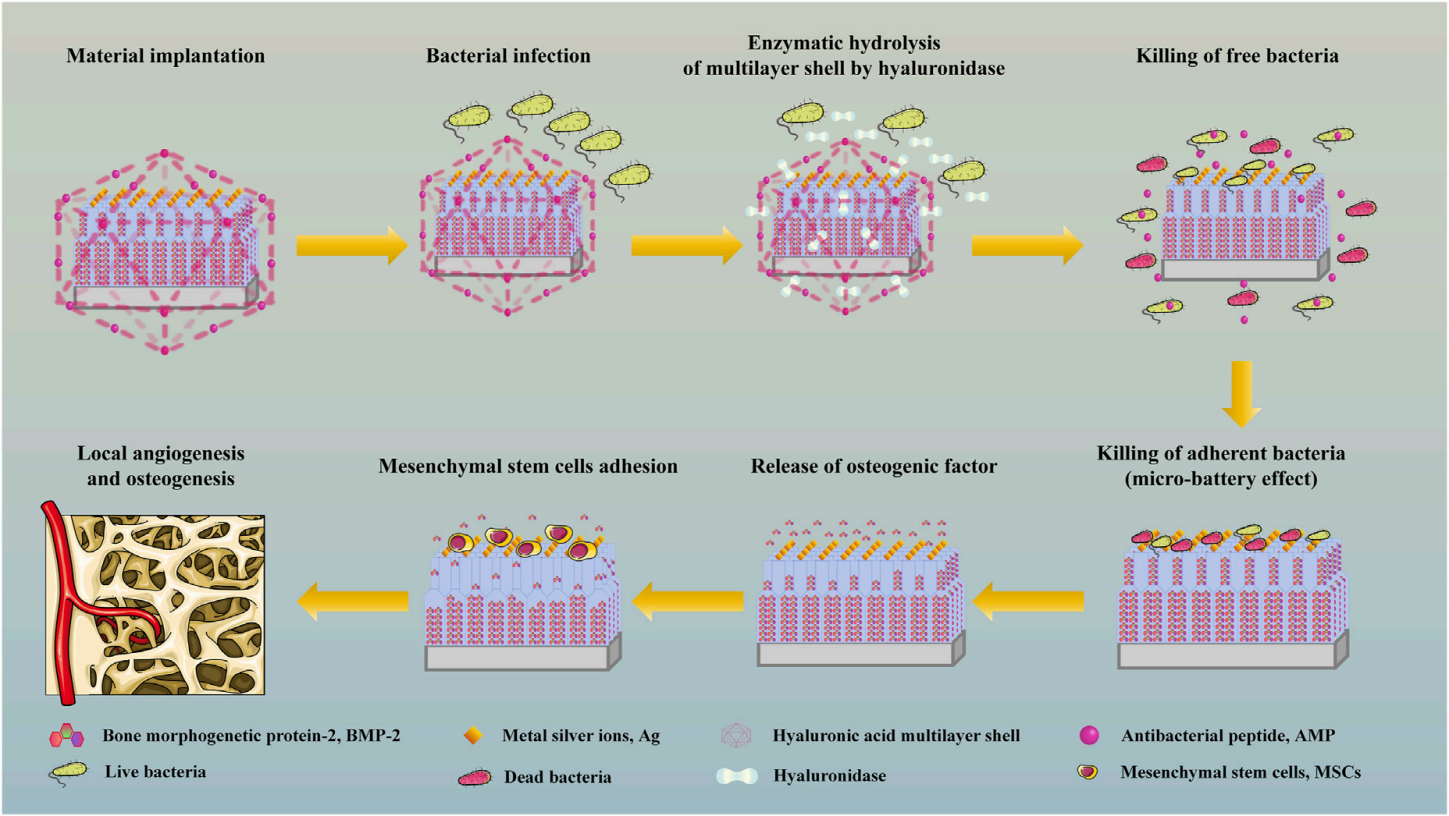
Mechanism diagram of antibacterial scaffold material after implantation.

TiO_2_ nanotubes are prepared on the surface of Ti by anodic oxidation and construct a double-layer nanotube structure, and osteogenic drugs are filled into the nanotubes by vacuum-assisted physical adsorption, so as to realize long-term sustained release of osteogenic drugs and promote continuous osteogenesis. Metal silver ions (Ag) are deposited on the surface of the material by sputtering, which makes the implanted material have higher specific surface area, produces electron biological effect, realizes *in situ* sterilization and promotes angiogenesis. The outer layer of the material is prepared from covalently grafted antibacterial peptide (AMP) and hyaluronic acid (HA) by layer-by-layer assembly technology (LBL). When local bacterial infection occurs, the multilayer shell will be hydrolyzed by enzymes secreted by bacteria, thus releasing AMP to kill free bacteria, produce immune regulation and repair tissue damage (Figure 1). The antibacterial material has a dual sterilization response system of electronic biological antibacterial response and enzymatic hydrolysis antibacterial response, which can eradicate planktonic bacteria and adherent bacteria at the injured part after the material is implanted, assist the double-layer tubular structure to continuously and fully release osteogenic drugs, and assist the sputtering deposition ion structure with high specific surface area on the surface of the material to accelerate the formation of *in situ* bone tissue in multiple directions (Figure 2).

## Application and modification of Ti

Metal materials have good biocompatibility, strong corrosion resistance and excellent mechanical properties, so they are mainly used in parts that need mechanical support, such as bone defects of long bones. Metal scaffolds can effectively avoid extremely low intraosseous stress change and bone absorption between implants and cortical bones, thus promoting the growth of cells in the interface of implants and enhancing the bonding strength between bone tissues and implants (Dec et al., 2022; Bakhshandeh B. et al., 2017). The main problem of metal implant is that the corrosion of physiological environment will change the physical and chemical properties of the material, make the implant loose and damaged, and the increase of metal ion level will have potential toxic and side effects on the body. Currently, the most researched and widely used non-degradable materials are mainly Ti and tantalum, and the degradable metal scaffold material is mainly magnesium.

After Ti is implanted into the body, the surface will become the niche of the surrounding cells, and the niche is the place where the biological response events of the host and the surrounding cells and tissues are concentrated. However, due to the biological inertia on the surface of Ti, the effective integration of Ti with surrounding bone tissue is greatly limited (Fukuda et al., 2011; He et al., 2018). Therefore, how to biologically activate the inert Ti surface, enhance the long-term stability of Ti-based implants and promote the *in situ* osseointegration has become a huge challenge in the current research field. After Ti is implanted into the body, it faces two major challenges, aseptic loosening and bacterial infection. After Ti is implanted into the body, the process of effective bone repair and reconstruction between bone and graft surface is the positive result of the synergistic interaction and precise regulation of many cells and factors, which has many similarities with bone growth and remodeling and fracture repair (Fukuda et al., 2011; Panetta et al., 2010; Einhorn, 2006).

**FIGURE 3:**
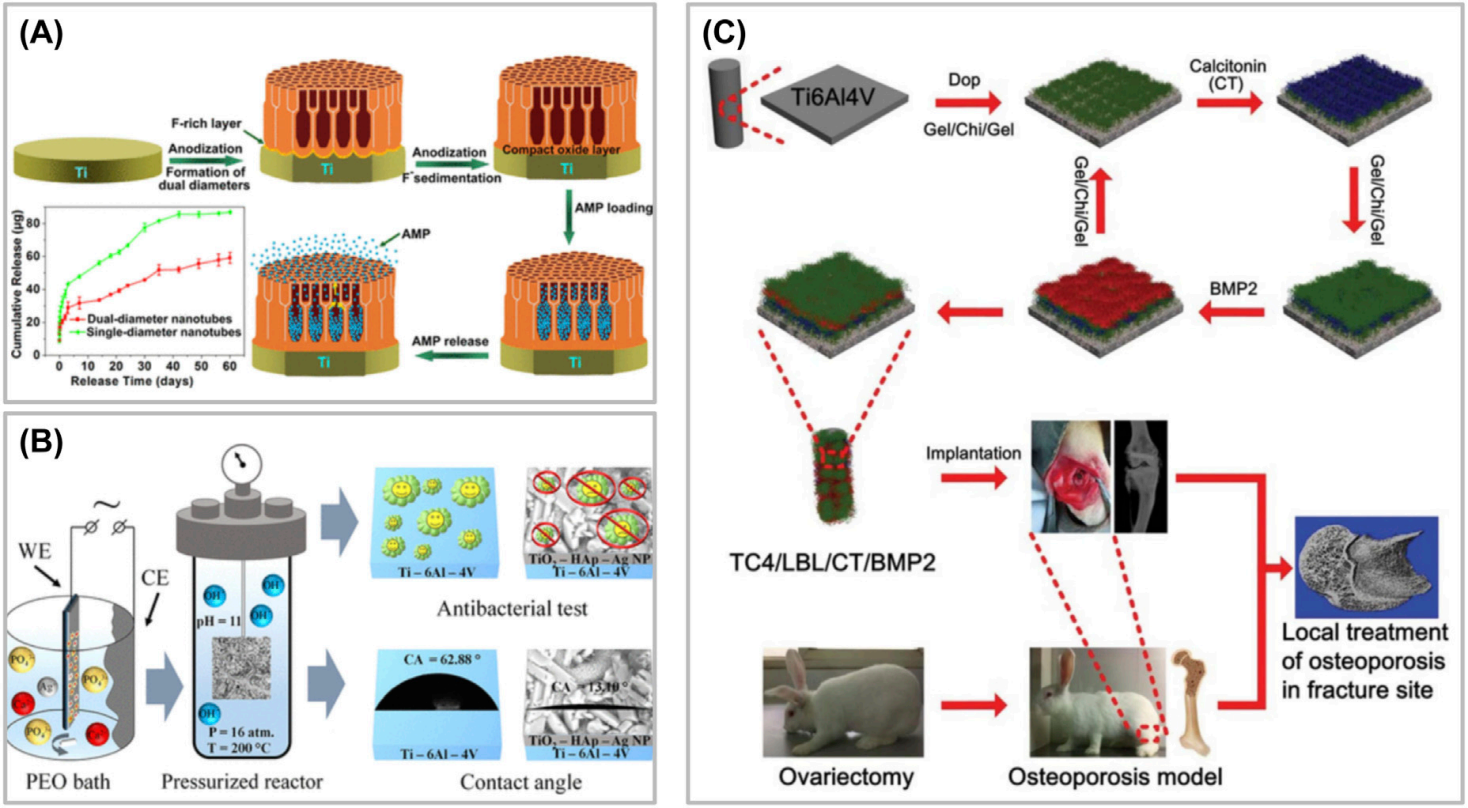
**(A)** Preparation of a double-layer nanotube drug storage pool system. **(B)** Preparation of Ti alloy system with Ag nanoparticles and calcium hydroxyphosphate by plasma electrolytic oxidation. **(C)** Schematic illustration of biofunctional multilayer coating on a Ti6Al4V (TC4) implant through layer-by-layer electrostatic assembly technique and investigation its regulation on local bone remodeling in the femur of an osteoporotic rabbit.

Brammer et al. (Brammer et al., 2009) prepared TiO_2_ nanotubes on the surface of Ti by anodic oxidation, and studied the pore sizes of nanotubes. The results showed that nanotubes with small diameters (less than 30 nm in diameter) were beneficial to cell adhesion, but not to cell differentiation. Large diameter nanotubes (between 70 and 100 nm in diameter) were not conducive to cell adhesion, but were beneficial to cell elongation, induced cytoskeleton stress to increase, and enhanced osteogenic differentiation. Zhang et al. (Zhang et al., 2017) prepared a double-layer nanotube drug storage pool system using Ti, with the upper nanotube diameter of 70 nm and the lower nanotube diameter of 140 nm, and loaded AMP by vacuum-assisted physical adsorption. The research results showed that the double-layer nanotube system could release drugs for up to 60 days, and could exert antibacterial activity for a long time without obvious cytotoxicity. To sum up, nanotube system and double-layer nanotube system have obvious advantages in sustained release and sustained action of drugs (Figure 3).

As a bone implant, solid Ti itself is dense and stable, and its elastic modulus is significantly higher than that of human bone, which will lead to stress shielding effect (Niinomi, 2008; Geetha et al., 2009). Under physiological conditions, the mechanical load can not be well transmitted from the implant to the surrounding bone tissue, which leads to bone absorption and movement at the material interface, and finally leads to the failure of material implantation (Tang et al., 2016). Porous Ti scaffold has a broad application prospect because of its suitable mechanical strength and a large number of coherent pore structures. Its large number of pores are beneficial to the circulation of body fluids, the transport of oxygen and nutrients and the formation of vascular tissue, and provide growth space for bone tissue. After the bone tissue grows into the scaffold, it forms a stable “mechanical lock” with the implant, which improves the interface bonding strength, reduces the stress shielding effect, and improves the long-term stability and life of the implant.

In the polysaccharide Ti-based system, the anti-adhesion polysaccharide Ti-based system often has high biocompatibility, and its degradation products are non-toxic and have good affinity with tissues. Anti-adhesion polysaccharide can form a physical barrier, reduce postoperative adhesion and produce significant anti-adhesion effect. At the same time, by adjusting the proportion of polysaccharide, the degradation rate of material coating can be changed to match the time of tissue healing (García-Robledo et al., 2024; Kasi et al., 2023; Pérez-Anes et al., 2015; Soylu et al., 2021; G et al., 2020; Ding et al., 2023; Tardelli et al., 2024; Xiong et al., 2025; Wennerberg and Albrektsson; Wang R. et al., 2022; Amirtharaj Mosas et al., 2022) (Table 2). Most polysaccharides have natural bacteriostatic effect, but their bacteriostatic effect is weak, so they are often combined with antibacterial components to achieve synergistic bactericidal effect to enhance antibacterial spectrum (Liu et al., 2025). At the same time, the sustained-release design of the reinforced material can prolong the antibacterial time of the material, thus reducing the number of times of administration. Some polysaccharides, such as carboxymethyl cellulose, have the ability to absorb exudate, maintain moist environment and promote healing (Ramezanzade et al., 2024; Osman et al., 2025; Wang et al., 2023). However, the mechanical strength of pure polysaccharide material is too low, so it needs to be compounded with Ti-based system to enhance its mechanical properties. At the same time, the antibacterial spectrum of materials dependent on polysaccharide type is limited, which may have poor effect on drugresistant bacteria, so at present, materials using polysaccharide alone are rare. For poly Ti without antibacterial drugs, it may have stable chemical properties and high-strength supporting ability, but its inert surface and no antibacterial property often lead to the lack of biological activity of the material. Polymeric Ti-based system loaded with antibiotics usually has strong antibacterial ability and local targeting effect, thus avoiding systemic drug side effects (Choi et al., 2024; Lv et al., 2024). However, the sudden release risk of drugs in this kind of materials may lead to insufficient drug concentration in the later period due to the sudden release of antibacterial drugs in the early stage, and the sudden release of drugs may lead to local toxic and side effects. Therefore, the optimization focus of this kind of materials is not only to improve the mechanical properties of Ti-based materials, but also to consider the drug release kinetics (Oliver et al., 2019).

**TABLE 2:** Comparison of anti-adhesion properties of materials.

Types of material system	Anti-adhesion effect	Anti-adhesion principle	Key influencing factors
Polysaccharide Ti-based	Excellent	Physical barrier, biological lubrication	Polysaccharide type, degradation rate
Unmodified polymeric Ti	Medium to poor	Surface roughness	The topological structure of Ti surface
Surface-modified polymeric Ti	Good	Coating reduces protein adsorption	Hydrophilicity and charge characteristics of coating

Ti-based orthopedic implant system is at the critical turning point of technological innovation, and the paradigm shift from “passive replacement” to “active repair” and from “single function” to “system intelligence” will be ushered in the next decade. With the cross-integration of materials science, bioengineering, nanotechnology and artificial intelligence, a new generation of Ti-based implant system will break through the traditional limitations and achieve a qualitative leap in bone integration efficiency, infection prevention and control and long-term service performance. Despite the broad prospects, the innovative development of Ti-based orthopedic implant system still faces multiple transformation barriers. The complexity of regulatory approval is the primary obstacle. FDA classifies smart drug release implants as “combined products”, which need to meet the double standards of drugs and medical devices at the same time. Also, the newly promulgated MDR regulations in the European Union put forward stricter biocompatibility requirements for nano-material implants, forcing enterprises to invest more resources in safety evaluation. Therefore, perfecting and innovating Ti-based orthopedic implant system as much as possible is a task that the entire orthopedic industry and even the industrial chain need to continue to develop and adhere to.

## Sustained release of osteogenic factors

The process of bone metabolism and repair after injury is highly complex, involving the interaction of various growth factors. Growth factors that can increase the growth activity of bone cells, regulate the growth and differentiation of bone cells and promote bone remodeling are called osteogenic factors. Osteogenic factors play an important role in the process of bone repair. Osteogenic factors can bind with target cell receptors and regulate various biological processes in cells (Tian et al., 2016). The process of bone repair after bone injury is a continuous process of bone repair-related cells such as osteoclasts and osteoblasts under the regulation of osteogenic factors. At the initial stage of bone injury, various osteogenic factors will be released, activating osteoblasts and osteoclasts, stimulating the proliferation and differentiation of osteoblasts, synthesizing bone protein and growth factors, and inducing bone formation (Q et al., 2017; Shirani et al., 2017). At present, there are many osteogenic factors studied, including bone morphogenetic protein (BMP), transforming growth factor-β, fibroblast growth factors, plateletderived growth factor and so on (Safari et al., 2021; Lowery and Rosen, 2018).

BMP is derived from bone and bone-derived cells and belongs to the transforming growth factor β superfamily. BMP is one of the most widely studied growth factors at present, and more than 20 BMP subtypes have been found, among which BMP-2, BMP-4 and BMP-7 have osteogenic ability, BMP-8 and BMP-9 are related to the formation of cartilage, and BMP-12, BMP-13 and BMP-14 are involved in the formation and repair of ligaments and tendons (Pallotta et al., 2018). BMP can participate in the expression of multiple signal pathways in the body and regulate the formation of cartilage, bone and blood vessels. BMP can activate Smad signaling pathway and mediate bone differentiation. BMP owns two kinds of transmembrane kinase receptors. When BMP binds to the transmembrane receptor on the cell membrane, it can activate Smad protein in cells, enter the nucleus, and activate downstream target factors such as osteocalcin, thus exerting its biological effects (Yoo et al., 2017; Zhao et al., 2016; Zhang et al., 2022; Lin et al., 2022). BMP has good bone induction ability, which can directionally induce MSCs to differentiate into osteoblasts, induce the proliferation and differentiation of various cells with osteogenic potential in bone tissue, synthesize collagen, promote the formation of cartilage and bone matrix, and form calcified bone tissue (Srouji et al., 2011), and owns the ability of cross-species induction of bone (Kloos et al., 2002). It has a good application effect on the treatment of fracture, bone defect and nonunion, and its clinical application in orthopedics has attracted extensive attention of researchers.

By vacuum-assisted physical adsorption, drugs promoting osteogenesis are filled into nanotubes, and appropriate solvents can be selected according to the physical and chemical properties of drugs, and the storage amount of drugs in nanotubes can be controlled. Controlling the degradation mode and rate of drug-coated materials can realize the degradation of materials under different response modes, thus realizing the release of osteogenic drugs and promoting local osteogenesis.

## The exertion of antibacterial effect

The incidence and economic burden of bone infection are shocking. The infection rate of joint replacement accounts for 0.3%–2.4% of total hip replacement and 1%–3% of total knee replacement (Qayoom et al., 2020). The most commonly used treatment for osteomyelitis is to fill the bone nest with polymethylmethacrylate cement impregnated with gentamicin (Gen) to eradicate bone infection. The main motivation of using bone active carrier to deliver antibiotics locally is to achieve controlled and sustained delivery of drugs at the infected site, so as to overcome the need for long-term systemic administration and induce the formation of bone structure at the surgically removed bone. In the osteomyelitis environment, the dead space produced by extensive debridement will be filled with hematoma and fibrous tissue, which will easily lead to fractures and further complications (Osmon et al., 2013). If the surrounding soft tissue capsule is damaged, the degree of vascularization will also be damaged, and the self-healing ability of bone tissue will be reduced, so it is very important to fill the residual dead space. Implants act as biocompatible and bioactive fillers in the dead space, which is helpful for the regeneration of bone tissue lost during surgical debridement. The implanted carrier can also be used as a drug carrier, which can maintain a high drug concentration locally, thus better managing infection, bone formation and defect healing. Using high concentration strategy to achieve satisfactory drug delivery and effective antibiotics to prevent infection may have side effects (Yu et al., 2022). Local antibacterial therapy is beneficial to treat local infection, improve bioavailability and deliver related substances to bone infection site in a targeted manner. Inflammation and impaired bone healing are common clinical symptoms of infection, and cytokines and growth factors secreted by attacked osteoblasts can also promote inflammation and thus affect bone regeneration.

According to the classification of antibacterial mechanism of implanted materials, the antibacterial function of materials can be divided into three categories: antibacterial adhesion, inhibiting bacterial contact and releasing antibacterial agents (Table 3) (Ran et al., 2018). The mechanism of antibacterial adhesion implant materials is to inhibit the adhesion of bacteria on the implant surface during the process of bacterial adhesion and biofilm formation. This antibacterial concept can be realized by improving the physical topology, biocompatibility and chemical groups of materials and drugs carried by materials (Kummer et al., 2013; Yu et al., 2015; Godoy-Gallardo et al., 2015). The mechanism of inhibiting bacterial contact implant materials is mainly to affect bacteria through the charge and chemical composition on the surface of the materials, thus affecting the physiological functions of bacteria such as enzyme activity and respiration, so as to achieve contact bacteriostasis (Chen et al., 2016; Lin et al., 2015; Yue et al., 2014). Special attention should be paid to the negative charge on the surface of bacteria, so it is a breakthrough for materials to exert antibacterial effect by using strong positive charge groups or quaternized polymers to interact with bacteria. Killing the pathogens around the implant by releasing antibacterial agents is the most commonly used strategy for releasing antibacterial implant materials, and the most commonly used way in clinic is to use antibiotics to inhibit or kill pathogenic microorganisms (Campoccia et al., 2010). AMP, a broad-spectrum antibacterial polypeptide, can physically penetrate and permanently destroy the bacterial membrane of bacteria, making them lose the integrity of structure and function, leading to the overflow of their contents, thus killing bacteria. AMP has good biocompatibility with eukaryotic cells, and has certain immune regulation and injury repair functions (Levy and Marshall, 2004; Spellberg et al., 2004). The antibacterial stability of AMP is affected by many factors, including structural characteristics, environmental conditions and microbial species (Zeng et al., 2016). For example, in the microenvironment of bacterial infection, the local environment is acidic, and the activity of AMP will increase in acidic environment, but it will decrease in neutral or alkaline environment, which just makes AMP play an effective bactericidal role under the condition of bacterial infection (Chen and Jiang, 2023). Effective combination of AMP and delivery system can not only protect AMP from degradation, but also realize sustained release of AMP and maintain local high concentration activity. Kazemzadeh-Nabbat et al. (Kazemzadeh-Narbat et al., 2010) electrodeposited micron-sized porous CaP coating on Ti surface, and then applied AMP to the surface, and found that the material had strong antibacterial effect. Most studies have confirmed that AMP plays an obvious role in local antibacterial of biomaterials.

**TABLE 3:** Classification of antibacterial function of materials.

Types of materials	Antibacterial mechanism	Mode of action	Antibacterial range	Potential problem
Antibacterial adhesion	Inhibiting bacterial adhesion	Physical barrier	Local	Failure caused by surface contamination
Inhibiting bacterial contact	Affecting bacterial function	Contact sterilization	Local	Material toxicity
Releasing antibacterial agents	Killing pathogens, destroying bacteria	Diffusion or active release	Regional	Drug resistance, difficulty in release control

Metal ions play an important role in bone tissue engineering. Metal ions not only play a significant role in promoting bone tissue growth, but also play an antibacterial role locally. Many studies have confirmed that metal ion modified composite scaffolds can effectively resist the infection of local bone defects and effectively promote bone regeneration (Lu et al., 2021; Wang F. et al., 2022; Kumagai et al., 2019; Yedekçi et al., 2021). Metal ion antibacterial agents have strong lethality to microorganisms such as bacteria. Nano-Ag was prepared *in situ* by plasma immersion ion implantation technology and embedded on the surface of Ti. The immobilized Ag in this system can overcome the toxicity caused by free Ag entering cells (Cao et al., 2011; Cao et al., 2013). Ions on the surface of Ti material can produce “micro-battery effect”, that is, electronic biological response occurs (Figure 2). The surface of bacteria is negatively charged, but there is no electron conduction chain on the surface of cells, so bacteria can play a charge attraction and aggregation reaction at the ions on the surface of materials without affecting the adhesion between materials and cells.

Ag nanoparticles have excellent broad-spectrum bactericidal efficacy, which can kill Gram-positive bacteria, Gram-negative bacteria, fungi and some viruses (Geng et al., 2017). Ag nanoparticles have great potential in the treatment of nonunion and bone defect caused by chronic infection. Because of its special sterilization mechanism, the related drug resistance events are rare (Rai et al., 2009). The combination of Ag nanoparticles and composite nanoparticles assembled from other materials can effectively reduce toxicity and maintain strong anti-infection performance. Ag nanoparticles are a broad-spectrum antibacterial agent, and their antibacterial stability is affected by chemical state, environmental conditions and carrier materials. Ag nanoparticles destroy microorganisms by destroying cell membranes, interfering with bacterial metabolism and producing reactive oxygen species, making it difficult to produce drug resistance (Guo et al., 2024). Free Ag nanoparticles have strong antibacterial activity, but they are easy to combine with anions to form insoluble precipitate, thus losing activity. The side effects caused by ion dissociation can be greatly avoided by fixing free Ag nanoparticles on TiO_2_ nanotubes caliber by sputtering spraying technology, and the cytotoxicity caused by excessive free ions can be greatly reduced. Sobolev et al. (2019) prepared a Ti alloy system with Ag nanoparticles and calcium hydroxyphosphate by plasma electrolytic oxidation, which could be transformed into silver sulfide nanoparticles *in vivo*, thus exerting anti-infection characteristics. Bakhshandeh S. et al. (2017) electrodeposited chitosan-gelatin composite layers doped with Ag and vancomycin on the surface of 3D printed porous Ti. In this system, vancomycin could be released continuously for at least 21 days, and played a synergistic antibacterial role, thus achieving the goal of completely eradicating planktonic bacteria and adherent bacteria, and there was no cytotoxicity. It has been pointed out that after cells are exposed to Ag nanoparticles, the expression of protein molecules related to bone cell pathway in stem cells, such as BMP-4, BMP-6 and FOS-like antigen, increases significantly (Qing et al., 2018), so Ag nanoparticles are often used as an important component to modify other materials because of their excellent antibacterial ability (Figure 3). Although ion doping endows bone repair materials with antibacterial ability, the sudden release effect of metal ions *in vivo* can not be overcome at present, and a large number of metal ions released in a short time have dose-toxic effects. Electroplating surface deposition technology can stabilize ions on the surface of materials, which is an excellent solution to solve the problem of sudden release of ions. In silver ion sputtering deposition, particle size is an important parameter to control the characteristics of materials. By adjusting the parameters of silver ion sputtering, controlling the target material and gas, and monitoring the surface in real time, the ion size can be accurately adjusted, so as to directionally design the material function. It can effectively reduce the problems of particle agglomeration and uneven edge effect (Kelly et al., 2003). HA, as the material of LBL, has normal degradation behavior after HA crosslinking for 4–8 weeks, which is just conducive to the release of BMP in TiO_2_ nanotubes to play a role in promoting bone, while Ag ions on the surface continue to play an antibacterial role (Lin H. et al., 2019). In the case of bacterial infection, bacterial secretions quickly act on LBL coating to achieve the bactericidal effect of AMP, improve the local infection environment, and facilitate tissue repair and osteogenesis.

## Application of LBL

There are many ways for local drug delivery. No matter which way is used to achieve effective local drug delivery, the ultimate goal of sustained antibacterial implant system is to achieve local sustained and stable drug release. The existing implant drug delivery technologies can use thin films (Rakib et al., 2022), hydrogels (Meng et al., 2023), microparticles (Kim et al., 2024), nanoparticles (Tengjisi et al., 2022), electrospun fibers (Krysiak et al., 2023) and TiO_2_ nanotubes (Xu et al., 2016), which have different characteristics and can be used in different scenarios (Table 4).

**TABLE 4:** The characteristics of various drug transport coatings and features.

Types	Drug release mechanism	Features	Disadvantage​	References
Thin films	Diffusion control, film degradation release	Simple preparation and soft fit	Low drug loading, poor mechanical strength, easy to break	Rakib et al. (2022)
Hydrogels	Swelling, shrinking response	High biocompatibility, adaptability to irregular tissues intelligent response	Sudden release effect, low mechanical strength rapid degradation	Meng et al. (2023)
Microparticles	Diffusion, polymer degradation	Protecting drug stability, targeted delivery	Complicated preparation process, causing local inflammation	Kim et al. (2024)
Nanoparticles	Environmental response, evolutionary power reactors	High permeability, surface modification	Potential toxicity, difficult to prepare a lot	Tengjisi et al. (2022)
Electrospun fibers	Fiber degradation, drug diffusion	High specific surface area Simulating ECM structure	Uneven distribution of drugs, different mechanical properties	Krysiak et al. (2023)
TiO_2_ nanotubes	Electrical stimulation, diffusion controlled release	High mechanical strength, controlled release, large drug loading	Bioinert surface	Xu et al. (2016)

LBL is a kind of technology based on polyelectrolyte multilayer films formed by alternating adsorption and deposition of oppositely charged polyelectrolytes through electrostatic action. With this technology, many proteins, drugs and so on can be encapsulated in materials to regulate cell behavior (Figure 3) (Guo et al., 2017; Shen et al., 2016a; Huang et al., 2016). The surface polyelectrolyte multilayer films prepared by LBL are similar to natural ECM, and have the characteristics of strong biocompatibility and are beneficial to cell adhesion, proliferation and differentiation. At the same time, the degradation of multilayer films can release the drugs wrapped in them, thus further stimulating and regulating the biological behavior of cells. LBL has been widely used in the preparation of bone repair materials because of its simple preparation technology and simple operation process. At present, chitosan is the most common medium for LBL. Chitosan widely exists in ECM, which is one of the main components connecting collagen fibers. Chitosan can provide microenvironment for cell proliferation and ECM production, and has the potential to promote osteogenesis (Zang et al., 2017). Chitosan not only has good biocompatibility, safety and low antigenicity, but also has antibacterial and antifungal properties, which can inhibit the reproduction of bacteria, fungi and viruses. Therefore, chitosan is often used as an antibacterial material in combination with other materials to treat infectious bone defects. Because simple chitosan has weak mechanical properties, poor water solubility and low biodegradation rate, chitosan is often used as scaffold coating or modified by chemical modification to prepare injectable hydrogel, giving full play to its advantages. Shen et al. (2016b) used silkworm-free peptide to load TiO_2_ nanotubes and covered them with LBL multilayer structure with silkworm-free peptide/chitosan coupling HA, which could achieve short-term and long-term antibacterial effect of hyaluronidase triggered by potential bacteria. Studies have shown that the HA-Gen derivatives with hyaluronidase response were formed by covalently grafting Gen onto HA molecules, and then the multilayer membrane structure of chitosan/HA-Gen derivatives was constructed by LBL to encapsulate the nanotube storage pool of deferoxamine drugs. During local bacterial infection, a large number of hyaluronidase secreted by bacteria could hydrolyze HA-Gen derivative multilayer layers, which on the one hand released Gen fragments for sterilization, and on the other hand released deferoxamine in nanotubes to promote bone formation and angiogenesis, thus accelerating bone repair.

After the antibacterial osteogenesis system constructed in this study is implanted, LBL coating will not be hydrolyzed by enzyme in a non-infectious environment, achieving the effect of gradual decomposition, and slowly released AMP will be gradually metabolized. At the same time, the release of BMP in nanotubes will promote local bone repair, thus achieving the effects of preventing infection and promoting bone regeneration. In the case of local bacterial infection, LBL coating is hydrolyzed by enzyme, which quickly hydrolyzes and releases a large amount of AMP for antibacterial effect. At the same time, Ag nanoparticles sputtered on the surface of nanotubes achieve continuous antibacterial effect, and BMP in nanotubes will also be released to accelerate local bone integration, thus realizing the organic combination of antibacterial and promoting bone. The loading amount of the drugs used should be strictly controlled, so as to achieve effective antibacterial and bone-promoting effects, and at the same time, it will not produce additional toxic and side effects on cells.

## The challenge of the antibacterial drug delivery system

The purpose of this study is to develop a material: Ti-based bone repair material with dual-response antibacterial system and sustained drug. The purpose is to prevent infection and promote local bone repair under the condition of no local bacterial infection after the implant is implanted in the body, and to respond to bacteria and accelerate local bone repair under the condition of local bacterial infection. The existing technology and materials can completely realize the expected construction of the above materials, but the main challenge is to find the best composition ratio of bone factor and antibacterial components loaded in the implanted materials, and to strive to achieve the maximum drug loading of the two drugs without mutual influence, and at the same time, the drug content has no toxic effect on the body, and to play the most lasting and effective osteogenesis and antibacterial role. Under the exploration of basic experiments, this study should not only overcome the above problems, but also explore the relationship between antibacterial effect and osteogenic effect after implantation of this material as much as possible.

## Conclusion

The application of materials provides a scaffold for bone regeneration-related cell growth for bone defect repair, and also helps to maintain the integrity of bone structure with its own mechanical properties. In this hypothesis, the Ti scaffold with double-layer nanotube structure with slow-release osteogenic factors not only provides a niche for osteoblasts to adhere, but also stores osteogenic factors to the greatest extent and continues to play a slow-release role. The existence of the scaffold fills the gap conducive to hematoma formation and provides favorable conditions for further osteogenesis. As for antibacterial, the enzymatic antibacterial system in the scaffold can release AMP in the case of bacterial infection to eradicate free bacteria. The ion antibacterial system located on the surface of Ti scaffold can not only destroy adhesion bacteria, but also provide a rougher and broader site for cell adhesion, which provides favorable conditions for cell proliferation, differentiation and osteogenesis in the next step. At the same time, BMP-2 slowly released from the double-layer tubular structure has a strong osteogenic and angiogenic effect, which promotes the osteogenic efficiency of the system. This kind of Ti-based bone implant with sustained drug release and high specific surface area wrapped by LBL of dual-responsive antibacterial system has a strong development prospect. The preparation of the scaffold combines the cross-integration of material science, bioengineering and nanotechnology, breaks through the traditional limitations, and improves the optimization of Ti-based materials in bone integration efficiency, infection prevention and control and long-term service performance.

## Data Availability

The original contributions presented in the study are included in the article/supplementary material, further inquiries can be directed to the corresponding authors.
